# Recurrent spontaneous pneumothoraces and vaping in an 18-year-old man: a case report and review of the literature

**DOI:** 10.1186/s13256-019-2215-4

**Published:** 2019-09-09

**Authors:** Alex Bonilla, Alexander J. Blair, Suliman M. Alamro, Rebecca A. Ward, Michael B. Feldman, Richard A. Dutko, Theodora K. Karagounis, Adam L. Johnson, Erik E. Folch, Jatin M. Vyas

**Affiliations:** 1 Department of Medicine, Harvard Medical School, 25 Shattuck Street, Boston, MA 02115 USA; 2 Department of Medicine, Massachusetts General Hospital, 55 Fruit Street, Boston, MA 02114 USA; 3 Division of Pulmonary and Critical Care, Massachusetts General Hospital, 55 Fruit Street, Boston, MA 02114 USA; 4 Division of Infectious Disease, Massachusetts General Hospital, 55 Fruit Street, Boston, MA 02114 USA

**Keywords:** Primary spontaneous pneumothorax, Pulmonary disease, Vaping, Electronic cigarettes

## Abstract

**Background:**

Primary spontaneous pneumothorax is a common disorder occurring in young adults without underlying lung disease. Although tobacco smoking is a well-documented risk factor for spontaneous pneumothorax, an association between electronic cigarette use (that is, vaping) and spontaneous pneumothorax has not been noted. We report a case of spontaneous pneumothoraces correlated with vaping.

**Case presentation:**

An 18-year-old Caucasian man presented twice with recurrent right-sided spontaneous pneumothoraces within 2 weeks. He reported a history of vaping just prior to both episodes. Diagnostic testing was notable for a right-sided spontaneous pneumothorax on chest X-ray and computed tomography scan. His symptoms improved following insertion of a chest tube and drainage of air on each occasion. In the 2-week follow-up visit for the recurrent episode, he was asymptomatic and reported that he was no longer using electronic cigarettes.

**Conclusions:**

Providers and patients should be aware of the potential risk of spontaneous pneumothorax associated with electronic cigarettes.

## Background

Primary spontaneous pneumothorax impacts approximately 20 out of 100,000 males and 6 out of 100,000 females annually [1]. Pneumothorax is characterized by the accumulation of air in the pleural space, often caused by ruptured subpleural blebs or bullae [2–4]. Risk factors that contribute to the development of spontaneous pneumothorax include tobacco smoking, age (adolescents and young adults), thin stature, and male sex. Furthermore, recurrent spontaneous pneumothoraces occurs in 20–60% of patients [5]. Age, gender, tobacco smoking habits, pneumothorax size, low body mass index (BMI), and treatment modality have been suggested to contribute to recurrent spontaneous pneumothoraces [4, 6]. Although cigarette smoking is a well-established risk factor for spontaneous pneumothorax [7–9], little is known about the contribution of electronic cigarettes to the development of spontaneous pneumothorax.

The use of electronic cigarettes has rapidly increased over the past decade, in particular among adolescents and young adults. Approximately 20.8% of high school students and 4.8% of adults age 18–34 years currently use e-cigarettes (also known as electronic cigarettes) [10]. Electronic cigarettes (that is, vapes) are portable, battery-powered devices that heat a complex liquid mixture containing nicotine to produce a vapor that users inhale. Many devices rely on replaceable liquid “pods” that may contain propylene glycol, glycerol, benzoic acid, nicotine, and artificial flavors [11]. A recent study reported high nicotine concentrations of pods ranging from 21.8 mg/mL to 59.2 mg/mL. These pods have considerably higher levels of nicotine per puff than older generation electronic cigarettes [12]. The long-term adverse health effects due to vaping are not well known, but several case reports have associated vaping with adverse respiratory, cardiovascular, neurological, and gastrointestinal health effects [13–17]. Unfortunately, it remains unknown whether vaping is a risk factor for spontaneous pneumothorax. Here, we describe an 18-year-old man with recurrent spontaneous pneumothoraces within 2 weeks that were temporally correlated with vaping.

## Case presentation

An 18-year-old Caucasian man presented in January 2019 to our Emergency Department (ED) for evaluation of sudden-onset right-sided chest pain while sleeping. He reported waking up with acute right-sided pleuritic chest pain underneath his ribs with radiation to his right scapula, which was made worse with inspiration and movement. He denied classic triggers, including excessive coughing, recent respiratory tract infection, or trauma prior to the onset of pain. However, he reported multiple episodes of vaping daily and rare intermittent marijuana use, but denied cigarette smoking or use of smokeless tobacco. He had no past medical or surgical history, history of vision changes, heart problems, or joint laxity. In addition, there was no family history of Marfan syndrome or lung disease. He reported no current medications, cigarette smoking, or alcohol consumption.

A physical examination revealed a height and weight of 54.9 kg and 180 cm, respectively with decreased breath sounds over his right lung. His calculated BMI had an underweight BMI of 16.9 kg/m^2^. His vital signals were notable for a temperature of 36.4 °C, heart rate of 64, blood pressure of 112/59 mmHg, respiratory rate of 19, and oxygen saturation of 95%. A neurological examination revealed he was alert and oriented to person, place, and time, and he moved all extremities equally. The remainder of the neurologic examination was non-focal. Laboratory examinations were all normal (Table 1).

**Table 1:** Laboratory examination for the both admissions

	First admission	Second admission
Metabolic parameters		
Sodium (mmol/L)	139	140
Potassium (mmol/L)	3.6	3.8
Chloride (mmol/L)	101	105
Carbon dioxide (mmol/L)	27	23
BUN (mg/dL)	16	11
Creatinine (mg/dL)	1.06	0.91
Glucose (mg/dL)	96	98
Albumin (g/dL)	4.5	4.2
Bilirubin (direct) (mg/dL)	0.3	< 0.2
Bilirubin (total) (mg/dL)	1.6	0.7
Total protein (g/dL)	6.9	6.9
Alanine aminotransferase (SGPT) (U/L)	7	8
Aspartate aminotransferase (SGOT) (U/L)	17	18
Alkaline phosphatase (U/L)	55	46
Blood gases		
pH, venous	7.35	7.34
pCO_2_, venous (mmHg)	52	51
Complete blood count		
White blood cell count (k/μL)	8.43	5.42
Platelet count (k/μL)	192	189
Red blood cell count (M/μL)	4.66	4.65
Hemoglobin (g/dL)	14.7	14.5
Hematocrit (%)	42.7	42.7
Mean corpuscular volume (fL)	91.6	91.8
Red cell distribution width (%)	12.5	12.4
Blood differential – absolute		
Neutrophils	–	2.48
Eosinophils	–	0.13
Basophils	–	0.04
Monocytes	–	0.61
Lymphocytes	–	2.16

*BUN* blood urea nitrogen, *pCO*_*2*_ partial pressure of carbon dioxide, *SGOT* serum glutamic oxaloacetic transaminase, *SGPT* serum glutamic pyruvic transaminase

**Fig. 1:**
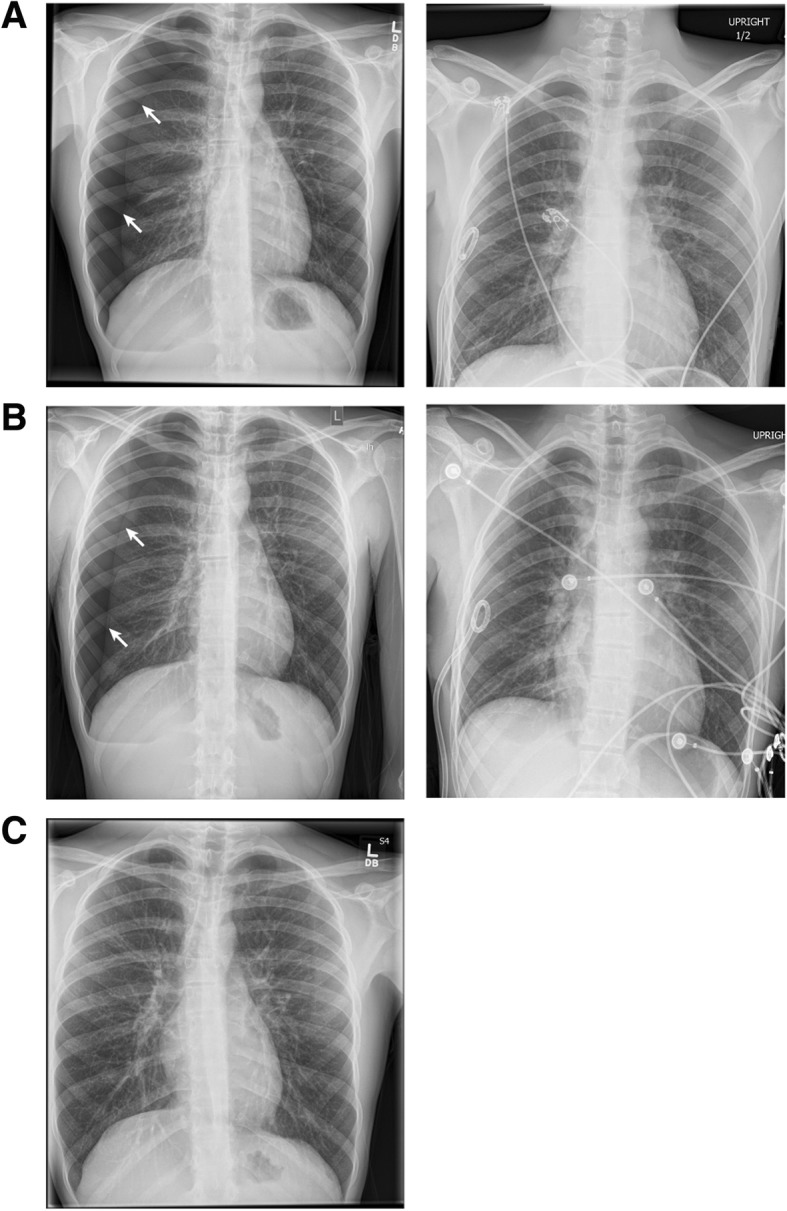
Chest X-ray at initial presentation demonstrating a right-sided pneumothorax (*arrow*) and resolving right-sided pneumothorax after pigtail chest tube placement for initial (**a**) and recurrent (**b**) spontaneous pneumothorax. (**c**) Sustained recovery from recurrent spontaneous pneumothorax two weeks after the removal of chest tubes

A chest X-ray (CXR) obtained in our ED demonstrated a large right-sided pneumothorax with evidence of tension (Fig. 1a). A chest tube was placed to suction with improvement in symptoms and our patient was admitted to the medicine service for further management. During admission, he received a lidocaine patch every 24 hours, acetaminophen (650 mg) every 6 hours, and ketorolac tromethamine (15 mg) every 6 hours, as needed. His pneumothorax resolved within the next few days and the chest tube was removed in a stepwise fashion. His presentation at the time was attributed to his body habitus. He was encouraged to quit vaping. He was scheduled for a follow-up CXR 2 weeks after discharge.

One week after discharge, he presented again to our ED for sudden-onset right-sided pleuritic chest pain and shortness of breath. Upon being admitted for recurrent spontaneous pneumothoraces, he reported daily vaping after discharge, but no fever, chills, hemoptysis, cough, upper respiratory infection (URI) symptoms, trauma, or recent airplane travel. At this time, he described a 1.5-year history of vaping with multiple devices. In addition, he again described occasional marijuana use, although marijuana had not been used between episodes of pneumothoraces.

On physical examination, his vital signs were within normal limits. His pupils were equal and reactive to light, without evidence of lens subluxation. His vital signals were notable for a temperature of 37.2 °C, heart rate of 63 beats/minute, blood pressure of 118/56 mmHg, respiratory rate of 18, and oxygen saturation of 97%. A neurological examination revealed him to be alert and oriented to person, place, and time, and moving all extremities equally. The remainder of the neurologic examination was non-focal. Laboratory examinations were all normal (Table 1). He had normal heart sounds without murmurs, rubs, or gallops. A lung examination was notable for decreased breath sounds over the right posterior chest.

**Fig. 2:**
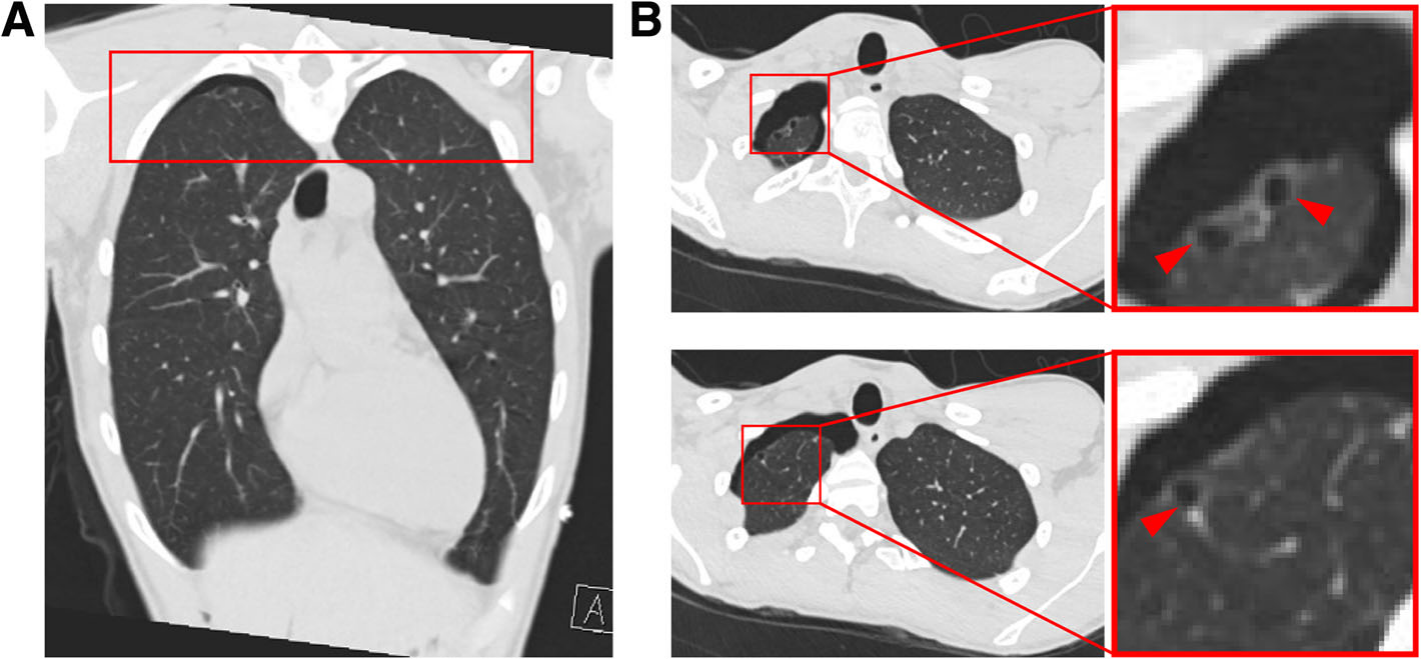
Coronal (**a**) and axial (**b**) computed tomography slices showing apical blebs and residual pneumothorax. Axial cuts selected from the *red box* in *panel A*. *Insets* from axial images highlight the presence of apical blebs (*arrowheads*)

A CXR revealed the presence of a large recurrent right-sided spontaneous pneumothorax without evidence of significant mediastinal shift (Fig. 1b). A follow-up chest computed tomography (CT) study without contrast after chest tube placement was notable for a small right residual pneumothorax with residual subsegmental atelectasis in his right lung and small right apical blebs (Fig. 2). The spontaneous pneumothorax was recategorized as secondary after noting apical blebs. He was treated with a pigtail chest tube after the initial CXR determined the presence of the spontaneous pneumothorax. His chest tube was removed when imaging confirmed durable resolution of the pneumothorax. His lungs were clear to auscultation bilaterally with symmetric breath sounds. During this second admittance, our patient was treated with lidocaine patch every 24 hours, acetaminophen (650 mg) every 6 hours as needed, and ibuprofen (600 mg) every 6 hours, as needed. A follow-up CXR 2 weeks after removal of the chest tubes confirmed resolution of the pneumothorax (Fig. 1c). At his follow-up appointment, he reported that he had quit using all e-cigarette products. A physical examination at this follow-up observed a temperature of 37.28 ºC, heart rate 79, blood pressure 110/72, respiratory rate of 20, and oxygen saturation of 96% on room air. On physical examination, he was in no acute distress with lungs clear to auscultation bilaterally and good excursion. No rales, wheezes, or rhonchi were noted on examination. In alignment with British Thoracic Society (BTS) guidelines [18], he was offered chemical pleurodesis and/or surgical options; however, he declined the procedure. Written informed consent was provided by our patient for the publication of this case report.

## Discussion and conclusions

Here, we present a case of an 18-year-old patient who presented to our hospital for recurrent right-sided spontaneous pneumothoraces in which each episode occurred within 2 weeks. He reported a history of vaping prior to the onset of each pneumothorax. The pneumothorax was resolved following pigtail chest tube placement. To the best of our knowledge, this is the first report to correlate recurrent pneumothoraces and vaping.

The potential adverse health effects of vaping have not been established, despite their growing use [19, 20]. Previous case reports have suggested that vaping is associated with multiple pulmonary processes including exogenous lipoid pneumonia, bilateral pleural effusion, bronchiolitis, acute eosinophilic pneumonia, and acute hypersensitivity pneumonitis [13, 21–24]. Here, we report a correlation between vaping and recurrent spontaneous pneumothoraces for the first time.

We hypothesize that electronic cigarette use could potentially increase the risk of spontaneous pneumothorax through the action of both inhaled toxins and through physiologic mechanisms. In marijuana smokers, the increased risk of spontaneous pneumothorax is thought to be partially attributable to deep inhalation followed by a Valsalva maneuver during exhalation [25]. Repeated deep inhalation through a highly resistive device, such as a water pipe, creating a Muller maneuver, and generating large negative intrathoracic pressure, has also been proposed as a mechanism of spontaneous pneumothorax in marijuana smokers [26]. Given the airflow resistance of pod-based electronic cigarettes, such as the ones used by this patient, there is certainly potential for a vaping-related Muller maneuver driving spontaneous pneumothorax in this patient. In fact, airflow rates of vaping apparatuses vary from 20 to 102 mL/s [27].

In addition to the potential physiologic risks of vaping, the nicotine and non-nicotine compounds inhaled in electronic cigarette smoke are associated with cellular injury and immune cell activation. Our patient reported using a JUUL device with mint flavored pods in the days leading to both pneumothorax episodes. In sampling multiple electronic cigarette delivery systems, JUUL pods were the only product to demonstrate *in vitro* cytotoxicity from both nicotine and flavor chemical content, in particular ethyl maltol [28]. The same study found that other marketed refill fluids can have a wide range of flavor concentrations, some as high as 362.3 mg/mL. Furthermore, vape liquid pods may contain numerous other compounds and are known to provide unreliable nicotine delivery that is often inconsistent with the labeling [29]. These liquid pods also contain propylene glycol, which has been shown to induce airway epithelial injury and deep airway inflammation [30].

Changes in “puff topography” as defined by puff volume, airflow, and duration can alter the chemical compounds delivered to the patient [27]. Heating vape liquid can alter the nicotine and chemical flavor content delivered [28, 31]. The flavoring chemicals can generate low molecular weight carbonyl compounds, such as formaldehyde and acetaldehyde, which are known carcinogens. The production of carbonyl compounds can increase with increasing concentration of flavor compounds [31]. Many vape pods contain the flavoring chemicals diacetyl and 2,3-pentanedione [32]. These two specific compounds have been shown to directly alter the transcriptional profiles of primary human bronchial epithelial cells grown at air–liquid interface, phenotypically leading to impaired ciliogenesis [33]. Electronic cigarette use can alter gene expression in the innate immune system of the airways, leading to increased levels of enzymes implicated in tissue injury including matrix metalloproteinase-9 and elastase [34]. The effect of vaping on the transcriptional profile and wound healing properties of the pleura remains unknown. Developing a full understanding of what chemicals are inhaled by someone who vapes remains challenging.

Although our patient reported no history of cigarettes or smokeless tobacco use, he did report a history of occasional marijuana use. Although a correlation has been made between marijuana and spontaneous pneumothorax, its causality remains undetermined as most studies were presented as case reports or small case series [35–37]. In a 2017 case-control study, daily cannabis smoking combined with tobacco use increased the risk of spontaneous pneumothorax compared to tobacco smoking alone in males [35]. It is possible that the combination of marijuana and vaping share similar effects and pathophysiology (that is, carbon monoxide burden and carcinogen additives) contributing to an elevated risk of spontaneous pneumothorax. In addition, marijuana smoking increases squamous metaplasia, airway lesions, goblet and basal cell hyperplasia, and cell disorganization [38]. Pre-existing changes to the airway due to marijuana use could further contribute to the susceptibility of spontaneous pneumothoraces in chronic electronic cigarette users.

Since his second admission to our hospital with recurrent spontaneous pneumothoraces, he has quit vaping and remained event-free 1-month post-discharge. Thus, our case report of a young adult with recurrent pneumothoraces correlated with vaping, to the best of our knowledge, is the first case report to associate spontaneous pneumothorax and vaping. We hypothesize that compounds delivered through the electronic cigarette may induce an innate inflammatory response in the lung and possibly alter airway and pleural gene expression to impair wound healing. Furthermore, the Muller maneuver performed when vaping through devices with relatively low airflow and high resistance may contribute to large transmural pressures across the alveolus increasing the likelihood of developing pneumothorax. In individuals who may have pre-existing blebs, these etiologies might increase the risk of pneumothorax. Chronic marijuana smoking has been proposed to lead to increased bleb formation, although proof of causality has been difficult to establish [39, 40]. It is possible that chronic vaping may also lead to bleb formation. Further studies are required to evaluate these hypotheses.

## Data Availability

Not applicable.

## Abbreviations

BMI: Body mass index
CT: Computed tomography
CXR: Chest x-ray
ED: Emergency Department
